# *Escherichia coli* O157:H7: distribution, molecular characterization, antimicrobial resistance patterns and source of contamination of sheep and goat carcasses at an export abattoir, Mojdo, Ethiopia

**DOI:** 10.1186/s12866-019-1590-8

**Published:** 2019-09-12

**Authors:** Solomon Abreham, Akafete Teklu, Eric Cox, Tesfaye Sisay Tessema

**Affiliations:** 1 Veterinary Drug and Feed Administration and Control Authority of Ethiopia (VDFACA), Veterinary drug registration, certification and administration directorate director, Addis Ababa, Ethiopia; 2 Department of Microbiology, Immunology & Veterinary Public Health, College of Veterinary Medicine and Agriculture, Debre Zeit/ Bishoftu, Ethiopia; 3 Faculty of Veterinary Medicine, Gent University, Salisburylaan 133, B-9820 Merelbeke, Belgium; 4 Institute of Biotechnology, Addis Ababa University, Addis Ababa, Ethiopia

**Keywords:** Abattoir, Antibiotic sensitivity, CT-SMAC, *E. coli* O157:H7, IMS, Latex agglutination, Multiplex PCR

## Abstract

**Background:**

Cattle have been identified as a major reservoir of *E. coli* O157:H7 for human infection; the ecology of the organism in sheep and goats is less understood. This study was carried out to determine prevalence, source of infection, antibiotic resistance and molecular characterization of *Escherichia coli* O157: H7 isolated from sheep and goat.

**Methods:**

Systematic random sampling was carried out at Modjo export abattoir, Ethiopia, from November 2012 to April 2013 to collect 408 samples from 72 sheep and 32 goats. Samples collected were skin swabs, fecal samples, intestinal mucosal swabs and the inside and outside part of carcasses as well as carcass in contacts such as workers hands, knife, hook and carcass washing water. Then, samples were processed following standard bacteriological procedures. Non-Sorbitol fermenting colonies were tested on latex agglutination test and the positives are subjected to PCR for detection of attaching and effacing genes (*eaeA)* and shiga toxin producing genes (*stx1* and *stx2*). All *E. coli* O157:H7 isolates were checked for their susceptibility pattern towards 15 selected antibiotics.

**Results:**

*E. coli* O157:H7 were detected in only 20/408 samples (4.9%). Among these 20 positive samples, 70% (14/20), 25% (5/20) and 5% (1/20) were from sheep, goats and knife samples, respectively. No significant associations were found between carcasses and the assumed sources of contaminations. Of all the 20 isolates virulence genes were found in 10 (50%) of them; 3 (15%) with only the *eaeA* gene and 7(35%) expressing *eaeA* and *stx2* genes. All the isolates were susceptible to Norfloxacin (NOR) (100%).

**Conclusions:**

The presence of virulence genes shows *E. coli* O157:H7 is a potential source of human infection in Ethiopia.

## Background

Currently, microbial food borne illness, caused by a wide spectrum of pathogens, is a global concern though extensive scientific progress and technological developments achieved in recent years. Most of microbial pathogens are zoonotic and have reservoirs in healthy food animals from which they spread to an increasing variety of foods. This makes foods of animal origin major vehicles of food borne infections [1]. Microbial contamination of meat may originate from the feces and skin of animals presented for slaughter and can be transferred to the carcass during skin removal and evisceration [2, 3].

*Escherichia coli* is a normal commensal microflora of the intestinal tract of animals and humans. In contrast, *E. coli* O157: H7, which is considered as a subtype of Shiga toxin-producing *E. coli* (STEC) strain, is known to cause human diseases as food borne pathogen and is determined by production of these virulence factors [3, 4]. The bacterium is known to cause the human illness such as haemorrhagic colitis (HC), haemolytic uremic syndrome (HUS), and thrombotic thrombocytopenic purpura (TTP) in all age groups, while children and elderly are more victims [5]. Intimin is responsible for the bacteria’s intimate adhesion to intestinal cells, causing the appearance of attachment lesions and erasure of the microvilli of the brush border of enterocytes. Intimin is encoded by the *eaeA* virulence gene [6]. Furthermore, the organism produces shiga toxin types1 and 2 (*stx1* and *stx2*) which are responsible for the death of intestinal, vascular, renal cells. They are encoded respectively, by the virulence genes *stx1* and *stx2*.

Cattle have been identified as a major reservoir of the organism and shed the bacteria in feces. The role of small ruminants as source of human infection through fecal shedding is being reported in a number of studies. So far, the majority of food poisoning outbreaks were traced to beef contaminated with *E. coli* O157:H7 [7, 8]. In this regard, the most common route of transmission has been reported to be raw or undercooked minced beef [7]. Nevertheless, varieties of other foods have also been implicated in causing outbreaks [9].

Outbreaks of *E. coli* O157 caused infections have been reported in different African countries, stretching from South to east and West parts of the continent [9–11]. However, there is limited data on the prevalence of the organism and its virulence gene diversity in ruminants, especially sheep and goats, and foods of animal origin in Ethiopia [12, 13].

Antibiotic use in STEC infections is controversial because of the potential to increase production and secretion of Shiga toxins [14]. However, increase in antibiotic resistance has been noted over the last 20 years [15–17].

The rising incidence and the potentially serious nature of *E. coli O157* infection are a cause for concern to public health authorities. In line with this, use of sensitive methods to detect *E. coli* O157 during investigations of outbreaks, surveillance and quality control are recommended [18].

In the presence of the above situations, very few attempts have been made to identify *E. coli* O157: H7 under Ethiopian conditions [12, 13]. Therefore, there is paucity of information regarding the prevalence, distribution, virulence characteristics and antibiotic resistance profile of *E. coli* O157: H7 in meat and abattoir house environments in Ethiopia. It has not yet been determined to what extent these environments serve as sources of *E. coli* O157: H7 particularly to red meat contamination. A study of such types would provide valuable information as to the major sites of contamination in abattoir environments and help in the implementation of strategies to minimize contamination levels.

## Materials and methods

### Study area

Lottery system was used to select the one in Modjo city from six (6) export abattoirs in the country for this study. The study was conducted from November 2012 to April 2013 at the export abattoir in Modjo town, Ethiopia. Modjo is the center of Lume District, eastern Showa administrative zone of Oromia Regional State, 73 km away from Addis Ababa, at an altitude of 1777 m above sea level. The average minimum and maximum temperature are 18^o^c and 28^o^c respectively [19].

Although there is seasonal variation, the abattoir slaughters 500–1500 goats every day and 200–600 sheep twice per week. The export abattoir where the study was conducted is well equipped with modern facilities and it is certified of International Organization for Standardization (ISO) 22,000. As an export standard abattoir, there is implementation of HACCP practices for maintaining hygienic standards of the abattoir. Sheep and goat are slaughtered separately but by the same personnel using `Halal` methods. All slaughtering operations are performed on overhead rails. The skins are washed by tap water; carcasses are washed by pressurized water, trimmed and stored in chilling room till transported to consumers. In the abattoir, there are clean areas for bleeding, dressing, evisceration and meat inspection. Animals slaughtered in the abattoir are exported to Middle East.

### Study animals

**Fig. 1:**
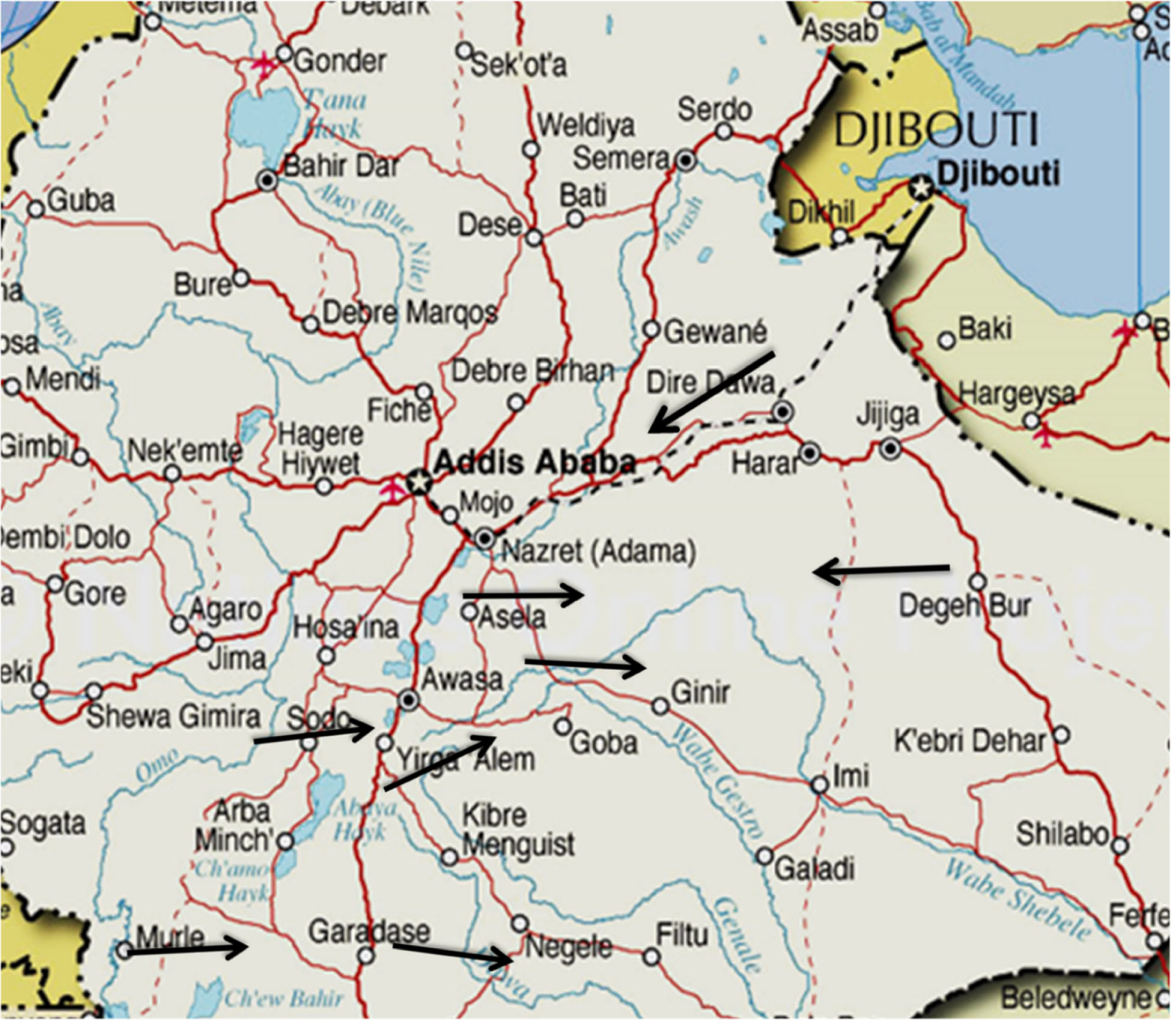
Catchment areas for sheep and goats slaughtered at the abattoir

The study was conducted on apparently healthy male sheep and goats slaughtered in the export abattoir during the study period. Animals are originated from different parts of the country mainly from Geanear (Bale), Somali, Awash-Metehara, Jima, Ambo, Borena, Arbaminch and Bati (Wollo). Most of them were transported to the abattoir by open aired vehicles, and this study considers these animals starting from the lairage (Fig. 1).

### Study design

A survey was conducted to determine the prevalence of *E. coli* O157:H7 on skin, feces, intestinal mucosal swab and carcasses of slaughtered animals and abattoir environment (carcass in contacts) particularly knives, water, hook and worker’s hand. Swab samples were collected from November 2012 to April 2013.

Systematic random sampling was used to select the sampled animals. Fecal samples and skin, carcass and intestinal mucosal swab samples were collected from each selected animal following their rail (tag) along the line of operation. Swab from abattoir environment, which are in contact with the carcass, were sampled once on each sampling day. Knives, hook, workers hands and the tap water were considered to be carcass in contacts. Water, which was used to wash the carcass, was sampled directly from the tap.

All samples were transported in icebox to Microbiology laboratory, College of Veterinary Medicine and Agriculture, Addis Ababa University (CVMA, AAU) and stored at 4 °C until processed. All samples were processed in 12 h interval. Culturing, isolation, identification and PCR were performed to determine the presence of *E. coli* O157:H7 in each sample and most effective drugs against the bacteria were selected after antibiotic sensitivity testing.

### Sample size determination

The number of study animals was determined based on the expected prevalence of *E. coli* O157:H7 and the desired absolute precision according to the formula stated on Thrusfield [20];

$$ \mathrm{n}={1.96}^2\ {\mathrm{P}}_{\mathrm{exp}}\left(1-{\mathrm{P}}_{\mathrm{exp}}\right)/{\mathrm{d}}^2 $$

Where:- n = required sample size; P_exp_ = Expected prevalence; d = desired absolute precision.

Based on a previous study done in Modjo and Debre-zeit export and municipal abattoirs, the prevalence rates of *E. coli* O157:H7 in goat and sheep were 2 and 2.5%, respectively [12]. Using these two expected prevalence, 95% confidence interval and 5% absolute precision; the number of sampled goats and sheep were estimated to be 31 and 38, respectively.

### Sample collection

**Fig. 2:**
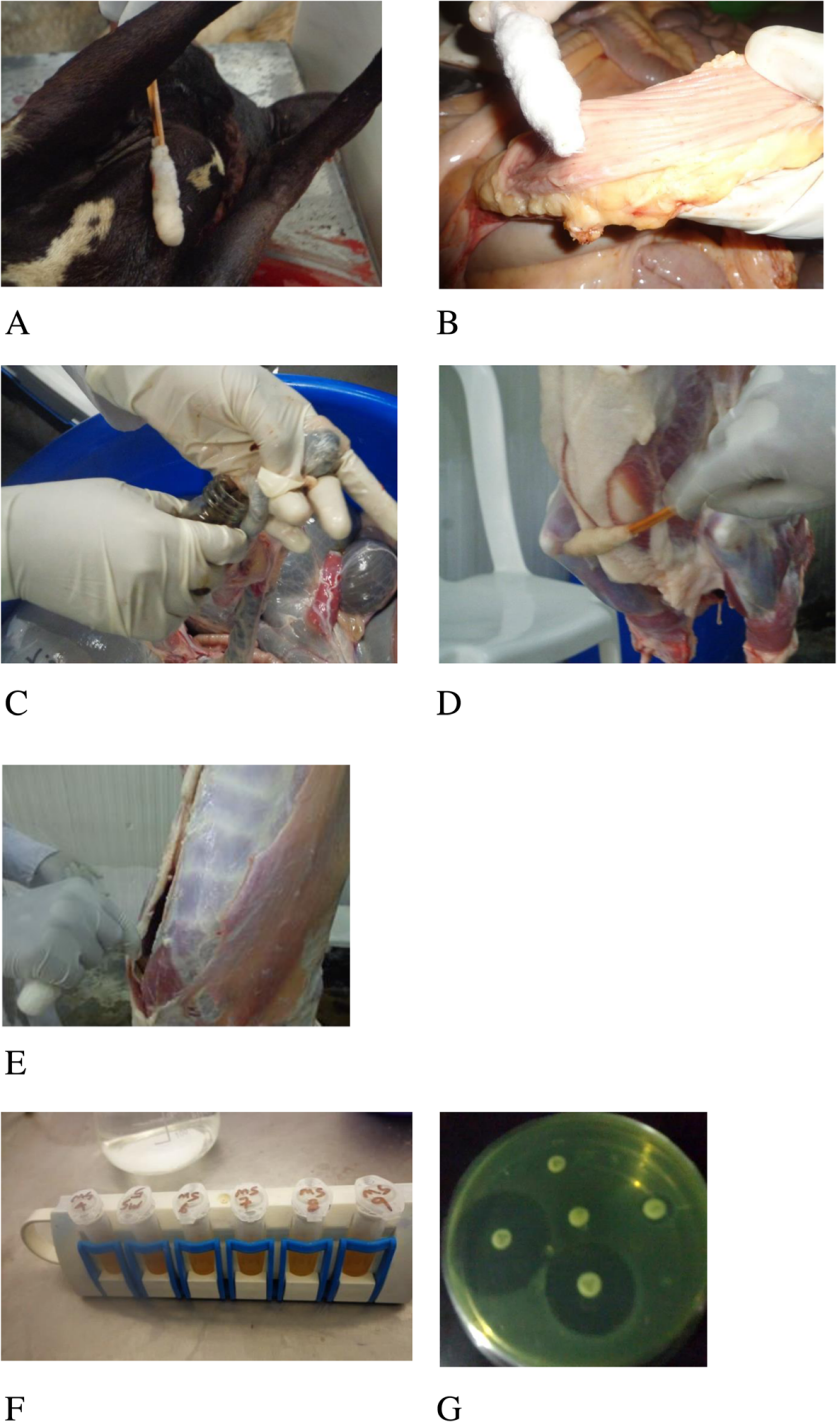
Procedures followed during sample collection and processing: **a** skin swab sample collection; **b** intestinal mucosal swab sample collection; **c** fecal sample collection; **d** carcass outside swab sample collection; **e** carcass inside swab sample collection; **f** concentration of *E. coli* O157:H7 using IMS technique; **g** antimicrobial susceptibility test bacterial isolates

Skin swab samples were taken according to McEvoy et al. [20], by using 2 × 3 cm sterile cotton tipped swabs soaked in approximately 10 ml of buffered peptone water (Oxoid Ltd., Hampshire, England). Skins were swabbed from the neck of animals over the line of bleeding before slaughtering near the bleeding area at an area of approximately 10 × 10 cm. Skin of the ventral midline part of the animal was also swabbed similarly at the mid line to determine its contact to the carcass during flaying (Fig. 2). The shaft of the swab was then broken by pressing it against the inner wall of the test tube and disposed. Finally this was repeated but with a dry sterile cotton.

Approximately 25 g of fecal samples were taken after complete evisceration directly by opening the rectum according to the method described by Elder et al. [21]. The whole abdominal digestive organs were separated from the slaughtering line in a plastic bucket and the rectum was opened using a sterile surgical blade (Fig. 2); the fecal sample was then put in to sterile universal bottle. Whereas for intestinal mucosal swab sampling, the distal colon was ligated and opened using a sterile surgical blade proximal to the rectum and the lumen was swabbed by using sterile swab. The swab was then introduced in to approximately 10 ml buffered peptone water in a sterile test tube.

The external parts of carcasses were swabbed from rump, midline and brisket area, just before chilling according to the methods described by McEvoy et al. [22]. Sterile cotton swabs soaked in approx. 10 ml of buffered peptone water was used to rub against the carcass first horizontally then vertically. A second dry sterile cotton swab was also rubbed at exactly the same area. For the internal part of the carcass, where contacts with other carcass is not possible, the thoracic and the pelvic parts of both sides, through the evisceration opening, were swabbed using the same procedure as above (Fig. 2). Disposable sterile gloves were used for each carcass and changed.

Similarly, abattoir utensils and other carcass in contacts such as knife, hook, water and workers hands were also swabbed as a sample using sterile cotton swabs soaked in approx. 10 ml of buffered peptone water. A pooled sample was taken from knives used for evisceration and carcass trimming on each sampling day. Only hooks used to hang the sampled carcass were also swabbed each sampling day as a pooled sample. Whereas abattoir workers whose hands have direct access to the washed carcass were swabbed for sampling, at their palm surface and fingers. Hand washing is practiced almost regularly. They wash their hands between each work which was practiced according to the abattoirs particular HACCP procedures. Moreover, 25 ml of water sample was collected directly from the tap which is used for washing of the carcass.

For each and every sampling a sterile latex glove was used to avoid cross contamination and every procedure was done as aseptic as possible. All samples were transported in ice box to the laboratory.

### Laboratory work

#### Bacteriological sample processing

Fecal samples were measured for accuracy and placed in to sterile stomacher bag and a 1:9 ratio of a modified tryptone soya broth (Oxoid Ltd., Hampshire, England) containing 20 mg|l novobiocin (Sigma, Steinheim, Germany) (mTSB+n) was added in it, and agitated in stomacher (Seward Stomacher 400, Seward, London, UK) for agitation at low speed for 30 s.

In to all other swab samples 90 ml of mTSB+n was added and homogenized using vortex mixer, and also for the 25 ml water sample 225 ml mTSB+n was added just to keep the 1:9 ratio.

#### Isolation and identification of *E. coli* O157:H7

Microbiological samples for the isolation and identification of this bacterium were processed as described as follows.

##### Selective enrichment

Modified tryptone soya broth containing 20 mg/l novobiocin (Oxoid, Ltd., Hampshire, England) was used at 1:9 ratios as mentioned above, for selective enrichment of all the samples. Then, all the samples types were incubated at 41.5 °C for 24 h.

##### Isolation by immuno magnetic separation (IMS) and culturing of the isolates

After 24 h of incubation all enriched broth culture were processed using IMS using Dynabeads anti-*E. coli* O157 (Dynal Biotech AS, ThermoFisher Scientific, Oslo, Norway) as follows. Both enriched broth culture and the paramagnetic beads were homogenized by vortexing and 1 ml of the enriched culture was put in to a sterile screw cupped eppendrof tube. A 20 μl of resuspended paramagnetic beads (Dynal Biotech AS, ThermoFisher Scientific, Oslo, Norway) was then transferred in to the same eppendrof tube, which was briefly vortexed on the dynal mixer (Dynal MX4sample mixer) (Dynal Biotech AS, ThermoFisher Scientific, Oslo, Norway) at 20 rpm for 30 min at room temperature, for the bacteria to attach to antibody surface on the beads. The tubes were then put in to the manual magnetic particle concentrator (MPC-S) (Dynal Biotech AS, ThermoFisher Scientific, Oslo, Norway) with the magnetic strip in place, inverted 3 to 4 times and left to settle for about 5 min. It was then gently rotated for the magnetic beads to concentrate at the back of the tube. The cap of the tube carefully opened and the supernatant was discarded by carefully aspirating it with sterile fine tipped pipette, without touching the back wall of the tube. Then magnetic strip was removed and 1 ml of phosphate buffered saline containing 0.05% tween 20 (PBST, Sigma chemicals Co, Saint Louis, USA) was added to each tube using another disposable fine tipped pipette. It was then inverted 3 times after the tubes were clothed, the magnetic strip replaced and the above step repeated at least twice. To prevent cross contamination the PBST was put in different small containers and for each sample and each material transferring new pipette tips were used. Finally the supernatant was aspirated; the magnetic strip was removed and about 100 μl of PBST was added in each tube and mixed gently [23].

Around 50 μl of IMS bead and bacteria complex were streaked onto Sorbitol MacConkey agar (Difco, Becton Dickinson, Claix, France) containing 0.05 mg/l cefixime and 2.5 mg /l potassium tellurite (Dynal Biotech ASA, Oslo, Norway) (CT-SMAC). Culturing was carried out carefully to obtain pure colonies and plates were incubated at 37 °C for 20–24 h. The CT-SMAC agar plates were examined for the presence of non-sorbitol fermenting colonies [24–27].

The non-sorbitol fermenting colonies on CT-SMAC appear as slightly transparent, almost colorless with a weak pale brownish appearance with a diameter of 1 mm [24, 25, 27]. Such colonies are sub cultured on CT-SMAC for further a confirmatory test.

Confirmatory test by latex agglutination.

Latex agglutination was performed for confirmation of *E. coli* O157:H7 using latex kit (ThermoFisher Scientific, Oslo, Norway). The latex kit consists of four components: latex test reagent, latex control reagent, the positive controls and negative controls. The test reagent is latex particles sensitized with specific rabbit antibody against O157 antigen and the control reagent consists of latex particles sensitized with rabbit globulin. The positive and negative controls are suspension of inactivated *E. coli* O157:H7 cells and inactivated non-specific *E. coli* cells respectively.

The test was performed according to the manufacturer instructions (Oxoid Ltd., Hampshire, England). But first the latex kit was checked for its performance by using the control suspensions in the kit, the test was continued after the positive control reacts with the test latex showing positive result. A drop of test latex and 0.085% sterile saline water were dispensed in to the reaction card separately. A few presumptive colonies (an average of 2 colonies) of *E. coli* O157 were taken and emulsified in to the saline water on the latex card, then slowly mixed with the test latex and checked for agglutination within 1 min. Isolates showing visible agglutination by reacting with the test latex solution are again sub cultured for virulent gene identification.

### Determination of virulence genes by polymerase chain reaction

Multiplex PCR was conducted to assess the presence of virulence genes (*stx1, stx2* and *eaeA*) in *E. coli* O157:H7 colonies, which were confirmed by latex agglutination, by using the methods described in Mora et al. [16] and Inat and Siriken [28]. DNA was extracted by boiling the isolates. Thus, each suspect colony was inoculated on CT-SMAC and incubated for 24 h at 37 °C to get fresh colony. Few colonies were then selected and suspended separately in 100 μl of sterile distilled water in eppendorf tubes; the suspensions were then boiled at 92.5 °C for 17 min in a water bath. After centrifuging at 13000 rpm for 10 min, the supernatant containing the template DNA was transferred into nuclease-free eppendorf tubes, and were stored at -20 °C until use.

Detection of the *stx1, stx2* and *eaeA* genes was performed according to the protocol indicated in Inat and Siriken [28] with slight modification. Thus, 2 μl of extracted DNA was used as a template in a reaction mixture with a final volume of 25 μl that contained 10 mM of each dNTP, 25 nM *stx1* primer, 25 nM *stx2* primers, 25 nM *eaeA* primer (Table 1), 1 U of Taq DNA polymerase (Qiagen, Hilden, Germany) in 1× PCR buffer and 2 mM of MgCl2. Amplification of DNA was conducted using initial denaturation at 95 °C for 3 min, 35 cycles of denaturation at 95 °C for 20 s, annealing at 58 °C for 40 s, extension at 72 °C for 1 min, and final extension at 72 °C for 8 min.

**Table 1:** Primers’ sequence used in multiplex PCR for amplification of *stx1*, *stx*2 and *eaeA* genes

Target gene	Primers sequence (5′-3′) (Forward/reverse)	Amplicon size (bp)	Reference
*stx*1	ATAAATCGCCATTCGTTGACTAC/ AGAACGCCCACTGAGATCATC	180	[27]
*stx*2	GGCACTGTCTGAAACTGCTCC/ TCGCCAGTTATCTGACATTCTG	255	[27]
*eaeA*	GACCCGGCACAAGCATAAGC/ CCACCTGCAGCAACAAGAGG	384	[29]

For gel electrophoresis, the 10-μl amplicon mixture was loaded onto a 1.5% agarose gel. Electrophoresis was conducted at 125 V for 1 h. A 100 up to 1000 bp molecular weight marker was used to identify the amplified products as a ladder, which was visualized by UV illumination.

### Antimicrobial susceptibility pattern

Antimicrobial susceptibility testing was performed following the standard agar disk diffusion method according to CSLI [30] using commercial antimicrobial disks (Oxoid Ltd., Hants, UK). The selected antimicrobials their symbols and inhibition zone size interpretations are listed in Table 2.

**Table 2:** Antimicrobials used, their symbols and inhibition zone size interpretation for Gram-negative enteric bacteria

Antimicrobial used ^a^	Symbols	Diameter of zone of inhibition in mill meter			
		Resistant ≤	Intermediate	Moderately Susceptible	Susceptible ≥
Amoxicillin (25 μg)	AML	13	–	14–16	17
Bacitracin (10 μg)	B	14	15–16	–	17
Cefotaxime (5 μg)	CTX	14	–	15–22	23
Cefoxitin (30 μg)	FOX	14	–	15–17	18
Ceftazidime (10 μg)	CAZ	12	13–17	–	18
Cefuroxime Sodium(5 μg)	CXM	14	–	15–22	23
Clindamycine (10 μg)	DA	14	15–20	–	21
Cloxacillin (5 μg)	OB	13	–	14–16	17
Doxycycline (30 μg)	DO	13	–	14–16	17
Kanamycin (30 μg)	K	13	14–17	–	1
Nalidixic acid (30 μg)	NAL	13	14–18	–	19
Nitrofurantoin (300 μg)	F	13	14–17	–	18
Norfloxacin (10 μg)	NOR	12	13–16	–	17
Polymyxin B (300 unit)	Pb	8	9–11	–	12
Vancomycin (30 μg)	VA	14	15–16	–	17

^a^all are Oxoid products (England)

Pure colonies, incubated for 6 h in Tryptone Soya Broth (Oxoid Ltd., Hants, UK) were processed to a turbidity of 0.5 McFarland standards (approximately 3 × 10^8^ CFU per ml) in a sterile saline solution. Then, they were inoculated on Muller-Hinton agar plates (Becton Dickinson company, Cockeysville USA) using sterile cotton swab, making sure that all the surface of the media is immersed with the bacterial suspension. Antibiotic discs (Oxoid Ltd., Hants, UK) were then dispensed and plates were incubated for 24 h at 37^0^c. Diameters of the zone of inhibition were measured and the results were classified as resistant, intermediate and susceptible according to CLIS [30]. *E. coli* ATCC 25922 type strains were used as a positive control.

### Data collection, management and analysis

The establishment of computer database and the necessary manipulations such as variable coding was performed using MS Excel (Microsoft® Excel® 2010, Microsoft Corporation; Santa Rosa, California, USA). The database was transferred to SPSS version 11.5 (IBM Corporation, New York, USA) [31] for analysis. Descriptive statistics such as proportions, standard deviations and 95% confidence intervals were performed. Over all and sample-specific prevalence were determined by dividing the number of positive samples to the total number of samples examined. Difference among and between proportions of the groups with certain determinant factors was determined by chi-square (**X**^**2**^) test. ORs were calculated using univariable logistic regression to determine the degree of associations of carcass contamination with fecal, skin and carcass in contact surfaces’ status. A *p*-value < 0.05 was considered indicative of a statistical significance difference.

Furthermore, Kappa statistics was performed to see whether there is agreement between fecal sample and intestinal mucosal as well as carcass inside and carcass outside swab *E. coli* O157: H7 status. Interpretation of the Kappa test was based on Rules-of-thumb for kappa: values less than 0.40 indicate low association; values between 0.40 and 0.75 indicate medium association; and values greater than 0.75 indicate high association between the two raters.

## Results

This study was conducted on 40 and 32 apparently healthy slaughtered sheep and goats, respectively, at an export abattoir, Modjo, Ethiopia, from November 2012 to April 2013. Bacteriological examination was conducted on fecal, skin, intestinal mucosal, carcass outside and carcass inside swab samples, and swabs from knife, personnel’s hand and hook as well as water samples. Carcass in contact samples were 12 each, since they were taken as a pool sample at each sampling day. Whereas, the other specific samples were taken from each animal, so were 72 each.

### Prevalence of *E. coli* O157:H7

From 408 samples examined for *E. coli* O157:H7 only 20 (4.9%) were found to be positive. It was present in feces, intestinal mucosal swabs, skins, inside and outside carcass swabs and knife used in the abattoir. From these 20 positive samples 70% (14/20) were from sheep and 25% (5/20) were from goats, the rest 5% (1/20) was from a knife.

Statistically significant difference (*p <* 0.05) was found in prevalence of *E. coli* O157:H7 between sheep and goats (Table 3). Due to low prevalence, small sample size and the abattoirs use of the same line for both species, complete separation of ovine and caprine for analysis was difficult. An animal was considered *E. coli* O157: H7 positive when it was positive for the bacteria on either fecal sample and/or intestinal mucosal swab; samples from fecal or intestinal mucosal swabs of goats were not positive. Skin, carcass inside, and carcass outside *E. coli* O157: H7 statuses were considered indicators of external contamination and were not used for the calculation of prevalence on animals. As a result, from the total 72 animals examined for the status of *E. coli* O157: H7, only 8 animals are considered positive. Statistically significant difference was observed between sheep and goat in harboring the bacteria; sheep being more prone to harbor *E. coli* O157:H7 than goats.

**Table 3:** Prevalence of *E. coli* O157: H7 by sample types and species of animals examined

Sample Type	Number of samples								
	Goats			Sheep			Total		
	Examined	Positives (%)	95% CI	Examined	Positives (%)	95% CI	Examined	Positives (%)	95% CI
Fecal	32	0 (0)	0–0	40	4 (10.0)	−0.39- 20.39	72	4 (5.6)	2.9–10.91
Skin Swab	32	3 (9.4)	−0.71-19.51	40	2 (5.0)	−2.55- 12.55	72	5 (6.9)	1.05–12.75
Mucosal Swab	32	0 (0)	0–0	40	6 (15.0)	2.63–27.37	72	6 (8.3)	1.93–14.67
Carcass outside	32	2 (6.2)	−2.16- 14.56	40	1 (2.5)	−2.91- 7.91	72	3 (4.2)	−0.43-8.83
Carcass inside	32	0 (0)	0–0	40	1 (2.5)	−2.91- 7.91	72	1 (1.4)	− 1.31- 4.11
Knife							12	1 (4.2)	−0.43- 8.83

*CI* Confidence interval

Of the 8 positive animals, only 2 (25%) were culture positive both for fecal sample and intestinal mucosal swab samples. The rest 6 (75%) were culture positive either for fecal sample or intestinal mucosal swab sample and were significantly different (*P* = 0.033). The agreement of the fecal sample and intestinal mucosal swab samples was measured using the Kappa statistics and the result indicated low agreement between the two (Kappa value = 0.357, 95% CI = 0.002–3.103) (Table 4).

**Table 4:** Comparative results by Pearson’s X^2^ test of species-specific *E. coli* O157: H7 prevalence in fecal sample and intestinal mucosal swabs

Sample type	*p-value*	Odds Ratio	CI for the Odds Ratio
Fecal	0.124	1.111	1.002–1.232
Mucosal swab	0.030	1.176	1.033–1.340

From all randomly selected 72 animals each type of samples were taken. Of the different sample types taken four fecal (5.55%), five skin swab (6.94%), six intestinal mucosal swabs (8.33%), one carcass inside swab (1.39%) and three carcass outside swab (4.17%) samples were found to be positive (Table 3). However, all the swab samples from personnel’s hand and hook as well as water samples were found to be negative for *E. coli* O157: H7.

The internal part of the carcass was also sampled to determine the impact of carcass contamination by feces especially during evisceration. It was thought to be helpful in avoiding the risk of contamination of the carcass by other in contacts, like washing water, hand etc. and evaluate only the risk of carcass contamination by feces during evisceration. Contamination of the inside part of the carcass will be from evisceration problems rather than external contacts and cross contaminations. If there were evisceration problems like opening the gut accidentally or others, it was believed that, contaminations will be at the midline and bottom part of the visceral part on the carcass. However, of all 72 samples of carcass inside samples taken during this study only 1(1.39%) was found to be positive for *E. coli* O157:H7 from sheep, with no significant importance.

The level of carcass outside contamination was considered as an outcome variable taking skin swab, fecal sample and knife swab as risk factors for carcass contamination. However, assessments using logistic regression analysis as well as Chi square test did not show significant associations between carcass contamination and the risk factors were (Table 5).

**Table 5:** Association of carcass outside contamination and *E. coli* O157: H7 status of the risk factors

Risk factors	*p-value*	Odds Ratio	95% Confidence interval for the Odds Ratio
Skin swab	0.999	0.928	0.868–0.991
Fecal sample	0.999	0.942	0.888–0 .999
Knife swab	1.000	0.986	0.958–1.014

Even if there is no association with carcass contamination in our finding, a higher prevalence of *E. coli* O157:H7 from skin swabs (6.94%) than from fecal samples of sheep (8.33%) was observed.

In the abattoir where these samples were collected, cold pressurized water wash was used to avoid visible contaminants such as blood clot, fecal debris, GIT (Gastro Intestinal Tract) contents so on. This was done after evisceration and inspection. The whole carcass surface was washed with this pressure wash. In addition to this, carcass trimming was performed using knives immersed in hot water after trimming of every carcass. The water was boiled at 100 °C, and used on open air, until changed with another batch of water. Carcasses are transferred manually by carrying from the overhead rails to the chilling room after weighing.

In addition, no positive carcasses were found from animals that were *E. coli* O157:H7 negative from their fecal, mucosal swab or skin samples.

Similarly, the level of carcass inside contamination was considered as an outcome variable taking skin swab, fecal sample and knife swab as risk factors for carcass contamination. However, such contamination sources were not significantly associated with carcass contamination and *E. coli* O 157: H7 status of the risk factors (Table 6).

**Table 6:** Association of carcass inside contamination and *E. coli* O 157: H7 status of the risk factors

Risk factors	*p-value*	Odds Ratio	95% Confidence interval for the Odds Ratio
Fecal sample	0.999	0.944	0.892–0.999
Knife swab	1.000	0.986	0.959–1.014

### Detection of virulence genes on the isolates

From the 20 *E. coli* O157:H7 isolates that were analyzed by PCR for the detection of virulence genes, 10 (50%) were found to be positive for having virulent genes. Among these 10 isolates, 7/20 (35%) of them are found to carry both *eaeA* and *stx2* and 3/20 (15%) carried only the *eaeA* genes; whereas no one of them carried the *stx1* gene (Table 7).

**Table 7:** Summary of virulent gene expression of *E. coli* O157:H7 isolates

Species of animals	Type of sample		Virulent genes expressed		
		*eaeA*	*stx1*	*stx2*	Both *stx2 & eaeA*
Sheep	Mucosal swab	4	–	4	4
	Fecal sample	1	–	1	1
Goat	Carcass outside	2	–	1	1
	Skin swab	2	–	1	1
	Knife	1	–	–	–
	Total	10	0	7	7

Out of the six isolates of the intestinal mucosal swabs of sheep four (66.7%) of them express the virulence genes, but only one of the four fecal isolates (25%) has these genes as a virulent factor. This shows the significance of *eaeA* being more prevalent in mucosal isolates than faecal isolates in this study. Of the caprine isolates both the carcass outside isolates show *eaeA* genes, but only one of them are with the *stx2*, whereas from the three isolates of skin swabs, two of them show *eaeA* and only one was with *stx2*. The only one carcass in contact isolate, from Knife was also found to express the *eaeA* virulent gene.

**Fig. 3:**
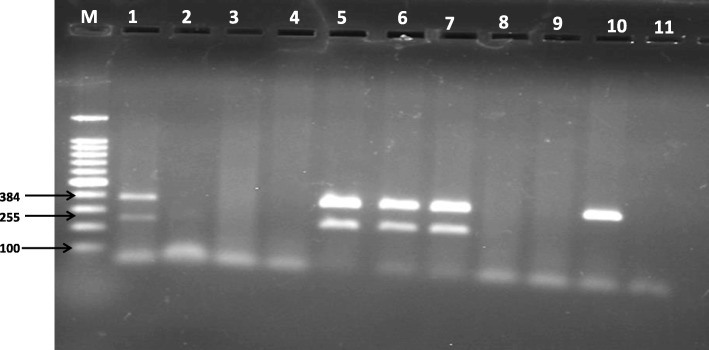
Amplification products of *eaeA* and *stx2* virulent genes of *E. coli* O157:H7 isolated from sheep & goat . M = 100 bp DNA marker; 1–10 PCR products, the amplicon sizes of *eaeA* and *stx2* are 384 bp and 266 bp, respectively; 11 = Negative contro, (PCR grade water)

In our finding seven of the isolates from goat and sheep had both *eaeA* and *stx2* genes and three of them were with only the *eaeA* gene (Table 7, Fig. 3), but none of them show the *stx1* gene.

### Antimicrobial susceptibility of the isolates

Testing of all the 20 *E. coli* O157: H7 isolates for fifteen different antimicrobials showed susceptibility to one of the antimicrobial used, Norfloxacin (NOR) (100%). However, another two antibiotics show growth inhibition zones for all isolates, even if two of the isolates (10%) are moderately susceptible for Ceftazidime (CAZ) and another completely different two isolates (10%) showed intermediate resistance to polymixin-B (PB). Another antibiotic showed growth inhibition zones on four of the isolates (20%) was Kanamycin (K), even if it was not enough to consider it as an effective drug (Table 8).

**Table 8:** Antimicrobial susceptibility pattern of *E. coli* O157: H7 isolates by species and sample type. N.B: All isolates was resistant to the rest of antibiotics tested in this study

Species of animal	Type of sample	No of isolates tested	Susceptibility Pattern								
			Norfloxacin			CAZ			PB		
			Sen. No. (%)	Int. No. (%)	Res. No. (%)	Sen. No. (%)	Int. No. (%)	Res. No. (%)	Sen. No. (%)	Int. No. (%)	Res. No. (%)
Goat	Skin	3	3	0	0	3	0	0	3	0	0
	CO	2	2	0	0	2	0	0	2	0	0
Sheep	Fecal	4	4	0	0	3	1	0	2	1	0
	Skin	2	2	0	0	2	0	0	2	0	0
	MS	6	6	0	0	5	1	0	6	0	0
	CO	1	1	0	0	1	0	0	1	0	0
	CI	1	1	0	0	1	0	0	1	0	0
	Knife	1	1	0	0	1	0	0	0	1	0
Total		20	20	0	0	18	2	0	18	2	0

*CO* Carcass outside, *CI* Carcass inside, *Sen.* Sensitive, *Res*. Resistant, *Int.* Intermediate resistant

## Discussion

Carcass contamination with *E. coli* O157:H7 may occur during slaughtering operations because of direct contact with contaminated materials such as skin, fecal material, knives, workers’ hands and the likes [2, 3].

### Prevalence of *E. coli* O157:H7

While cattle are generally regarded as the main reservoir of *E. coli* O157:H7 for human infection on other studies [8], the results of the present study indicate that sheep and goats may also be contributing sources, with sheep being more significant. This finding is in line with several previous studies which have indicated sheep as a reservoir of this bacteria [8, 32–35]. Nevertheless, it is not in agreement with a previous study in another export abattoir in Modjo, Ethiopia, which showed that both species are equally potential important sources of human *E. coli* O157:H7 infections [13]. This is may be due to the lack of the previous study to consider intestinal mucosal swab as a sample, since it shows the highest number of isolates in this study.

A 10% fecal prevalence of *E. coli* O157:H7 from sheep in this study was a little higher than previous studies conducted in this country and in other countries. Mersha et al. [13] reported a relatively lower (5.4%) prevalence of *E. coli* O157:H7 in sheep feces in the country. Similar low levels were reported in Netherlands [18], 4% in ewes and 4% in lambs, in Spain [32], 3% in lambs, in UK [6, 35], 1.7% in sheep in Great Britain [36], 1.4% in sheep, and in Italy [8], 0.2%. However, a much higher prevalence of 18% in Turkey [37], 31% in USA [38] and 68% from sheep flocks in Australia [39] from fecal samples were reported. On the other hand, zero *E. coli* O157:H7 prevalence from sheep fecal samples were reported in Norway [40], Scotland [41], Ireland [35], Greece [42] and United States [43]. Similarly, this study have smaller bacterial findings but on caprine. Closer results, however, are reported in Greece (1 out of 81) associated with human outbreaks from goats [42]. Similarly, Keen et al. [43] reported no *E. coli* O157:H7 from 526 goats’ in US zoological parks. In contrast, higher prevalence observed in other countries. A prevalence ranging from 55 to 95% reported in France [44] and 40% in Australia [45] in goats by flocks.

Marked differences in the prevalence of *E. coli* O157:H7 from fecal samples were observed in both sheep and goats. The variations in prevalence among the various studies could be due to different reasons such as variations animal management systems, geographical and climatic factors, sampling times and sampling technique, animal inherent factors, and so on. A number of studies have also shown that prevalence of *E. coli* O157:H7 shed from animal feces can vary significantly in relation to time, age of animals, nature of feeds etc. [3, 7, 25]. Moreover, Battisti et al. [8] indicated sheep husbandry as a possible reasons for *E. coli* O157 fecal prevalence differences in US as compared to European countries. In connection to this, a meta-analysis by Islam et al. [46] showed that world region, type of cattle breed and to some extent, specimens as well as method of pre-enrichment, were identified as factors for variation in the prevalence estimates of the organism in cattle worldwide. On the other hand, super-shedding animals may play unique roles in prevalence differences in various geographical regions as reported by McPherson et al. [47], where supper-shedding is indicated to contribute to contamination of hides in cattle, has also been reported in sheep. This could have an impact of the lower rate of detection of the organism in the current study.

The overall prevalence in skin swabs (6.9%) was comparable to the previous study done in export abattoir of Modjo, Ethiopia [13]. Isolation of *E. coli* O157:H7 from skin of these species is very rarely described. In Ireland as reported by Lenahan et al. [35], a 5.8% prevalence of *E. coli* O157:H7 from fleece, sampled by shaving was reported. However, in hides zero to 22% *E. coli* O157:H7 prevalence were reported from different sites of hides in US [3, 22].

*E. coli* O157:H7 prevalence of 4.17% from carcass outside swab in the current finding was comparable to a previous study done in east Showa of Ethiopia on meat samples of sheep and goat [12]. Lower prevalence than the present finding was reported in Ireland [35] and in England [7] that ranges between 0.7 and 4% from goats and sheep carcasses. Similarly, Chapman et al. [7] in UK reported 0.21, 1.22 and 1.17% prevalence of *E. coli* O157:H7 from minced meat, burger and sausages of lambs, respectively. On the other hand, much higher prevalence was reported from an export abattoir of Ethiopia by Mersha et al. [13], 8.7%, and in Australia by Sidjabat-Tambunan and Bensink [42], 29.2% (31/106) from sheep carcasses.

Differences in the reported prevalence could be due to differences in the method of sampling, number of samples, sampling sites, and so on. Thus, MacEvoy [22] recovered only one among the nine positive samples by swabbing but six by excision, showing sampling methods can be big factor. The surface swabbing method, which was used in this study, however, was also used by others [13, 29] and depending on the degree of abrasiveness the method is recommended as an alternative method since it is cost effective and nondestructive.

The internal part of the carcass was also sampled to determine the impact of carcass contamination by feces especially during evisceration. It was thought to be helpful in avoiding the risk of contamination of the carcass by other in contacts, like washing water, hand etc. and evaluate only the risk of carcass contamination by feces during evisceration. However, of all 72 samples of carcass inside samples taken during this study only 1(1.39%) was found to be positive for *E. coli* O157:H7 from sheep, with no significant importance.

The level of carcass outside contamination was considered as an outcome variable taking skin swab, fecal sample and knife swab as risk factors for carcass contamination. However, assessments using logistic regression analysis as well as Chi square test did not show significant associations between carcass contamination and the risk factors were (Table 5).

In contrast, the previous study on another export abattoir in Modjo showed a significant association with these risk factors except for carcass in contacts. This shows that the implementation of HACCP system in the abattoir has reduced the risk of contamination of carcasses during the slaughtering operation, as compared to the previous study with no HACCP system implemented. Even if no information is obtained about the slaughtering practice during previous study, as it was observed during this study the abattoir management and MoARD takes the credit for proper implementation of strict prevention methods and careful handling on the slaughtering line.

Results of this study were supported by other studies for the absence of association between hide prevalence and carcass contamination [21]. However, in those reports hide prevalence of *E. coli* O157:H7 was much lower than fecal prevalence. Possible explanations for this apparent discrepancy might be a difference in the selection of skin sampling site. A difference in the sampling site could affect isolation rate of a given pathogen. For example, Reid et al. [3] isolated 3.3% from rump, 4.4% from flank and 22.2% from brisket of *E. coli* O157:H7 by swabs on the same animals. This might be due to difference in the survival rates of *E. coli* O157: H7 in different sites of the skin of animals. Moreover, it has been indicated that one site on the skin may have higher levels of contamination than the others and, therefore, posing greater risks for carcass contamination.

On the other hand, associations of these risk factors with carcass contaminations were also reported from different parts of the world: associations with feces by Elder et al. [21, 48] and Griffin et al. [49]; and with skin by Reid et al. [3]. On the contrary, absence of association between hide prevalence and carcass contamination was reported by Elder et al. [21], and in those reports hide prevalence of *E. coli* O157:H7 was much lower than fecal prevalence. Possible explanations for this apparent discrepancy might be a difference in the selection of skin sampling site as mentioned above.

The higher prevalence of *E. coli* O157:H7 observed from skin swabs (6.94%) than from fecal samples of sheep (8.33%) disagrees with the report of Elder et al. [21], where they reported higher prevalence in feces (28%) than on hides (11%). The skin of animals could have a number of sources to carry *E. coli* O157:H7 such as the soil, feed, water, feces etc. but animals could shed *E. coli* O157:H7 seasonally in their feces and as a result might be negative for the organisms on sampling time. The skin of a given animal could be contaminated by fecal sources from themselves and other animals. Therefore, during transportation and in the lairage cross contamination of skins could occur due to a more close contact of animals and as result an increase in the apparent prevalence of *E. coli* O157:H7 on skin relative to feces could be observed.

Although pressure washing was applied on the carcass and also knives were immersed in hot water between trimming of successive carcasses, *E. coli* O157:H7 was isolated in one of the knives. This indicates that this could be a critical point for carcass contamination and the prevention methods should be applied strictly; in this aspect adequate temperature should be used for sterilization of knives. In addition, while transferring carcasses manually by carrying from the overhead rails to the chilling room, bacterial recontamination might occur and spread from one carcass to others.

In addition, no positive carcasses were found from animals that were *E. coli* O157:H7 negative from their fecal, mucosal swab or skin samples. This finding strongly suggests the presence of carcass cross-contamination in the abattoirs. Reducing the amount of *E. coli* O157:H7 in live sheep and goats will likely lower contamination not only of meat but also of other food and water supplies that are exposed to sheep and goat fecal matter.

Similarly, the level of carcass inside contamination was considered as an outcome variable taking skin swab, fecal sample and knife swab as risk factors for carcass contamination. However, such contamination sources were not significantly associated with carcass contamination and *E. coli* O 157: H7 status of the risk factors.

### Detection of virulence genes on the isolates

Shiga toxins, encoded by *stx1* or *stx2* genes, the pathogenicity island LEE, coding for factors causing for attaching and effacing lesions and the enterohaemolysin encoded by *ehxA* gene are the major virulence factors found in *E. coli* O157:H7 [50, 51]. In the current study, half of the positive isolates were having two of the virulence genes tested.

In our finding seven of the isolates from goat and sheep had both *eaeA* and *stx2* genes and three of them were with only the *eaeA* gene (Table 7), but none of them show the *stx1* gene. The recovery of *eaeA* positive organisms at higher rate in mucosal swabs than in fecal samples shows that the intimin gene encoded by *eaeA* helps the organism to adhere tightly to the intestinal mucosal. Thus, fecal samples are not as good as mucosal swabs in isolating the organism. The higher numbers of isolates of this study showing the virulence genes with a lower prevalence, as compared to only one isolate, in the previous study in this area, with higher prevalence, are needed to be given an attention. In addition, it indicates the dynamicity of the bacteria over years and the capability of it to evolve and adapt to new environments. In other studies undertaken in Greece [42] only *stx2* from a single *E. coli* O157:H7 isolate was obtained from goat feces*.* Of the total 33 isolates in France [35] from sheep, only five of the isolates carried the *stx1* and *stx2.* The identification of these virulence genes in this study from half the isolates indicates the potential of small ruminant carcass as source of *E. coli* O157:H7 for human infections in the country and its effect to the growing meat export market. It also suggests the need for further detailed epidemiological studies on *E. coli* O157:H7 in Ethiopia involving different export abattoirs and species of animals.

### Antimicrobial susceptibility of the isolates

This study showed complete susceptibility of all isolates to one of the antimicrobial used, Norfloxacin (NOR) and variable levels of resistance towards the other antibiotics tested. Hiko et al. [12], Faris and Mekonen [52] and Lula [53] have reported antimicrobial resistance patterns of *E. coli* O157:H7 isolates, from animal and human sources, in Ethiopia. Only three antibiotics namely Norfloxacin, Ceftazidime and Polymyxin B were seen to be effective against this bacteria but all the rest of the antibiotics used have no effect on it. A wider antibiotic resistance seen in this study is similar to other authors’ explanations about the bacteria [54–56]. This higher percentage of resistance might be due to presence of only few isolates tested for susceptibility compared to overall study population and variability of resistant gene within isolates for particular antimicrobials. Although there is difference in the sources of isolates, the 20 (100%) of present isolates resistant to one or more antibiotics were higher than 41% of those previously tested isolate by Mora et al. [16] in Spain from human, cattle, ovine and food. Moreover, the overall drug resistant in the present study is much more higher than those isolates tested by Wilkerson et al. [56], forty-four (6.6%) of 663 of bovine and 29 (12.2%) 238 of human *E. coli* O157: H7 isolates from feedlots in the mid-western United States and the Public Health Departments of Washington, Oregon, Nevada, Wisconsin, Georgia, and Illinois states. This could be due to difference in the type of samples, number of isolate and genetic variation of the isolate among different geographical areas. Whereas the susceptibility of all the isolates to Norfloxacine, and 18 of them for polymixin B and Ceftazidime encourages hope for finding the most effective antibiotic therapy against *E. coli* O157: H7.

## Conclusions

In conclusion, *E. coli* O157: H7 was detected from the feces, skin, intestinal mucosal swab and carcasses of sheep and goats and knife at export abattoir. This pathogen was isolated from carcasses, from the inside and outside parts that indicated the presence of carcass contamination during slaughter operations. Thus, interventions to reduce the occurrence of *E. coli* O157:H7 and reduce carcass contaminations were not absolute even if the prevalence are lower in this study. Prevalence of *E. coli* O157:H7 is higher in sheep than goats with statistically significant difference. The presence of *eaeA* and *stx2* positive *E. coli* O157:H7 in sheep and goats carcass should be given attention. Thus, in addition to cattle, sheep and goats could serve as source of human infection.

## Data Availability

Not applicable
